# BRAF V600E/TERT Promoter Co-Mutation Is Associated with Radioiodine-Refractory Differentiated Thyroid Carcinoma: A Translational Molecular Biomarker Study

**DOI:** 10.3390/biomedicines14071634

**Published:** 2026-07-20

**Authors:** Madina A. Mussulmanova, Laura A. Pak, Aidana M. Rakhmankulova, Arailym Baurzhan, Lyudmila M. Pivina, Diana A. Pak, Zhandos K. Burkitbayev, Andrey Yu. Orekhov, Azhar S. Baktiyar, Saltanat O. Bolsynbekova, Masahiro Nakashima

**Affiliations:** 1 Department of Clinical Oncology, Radiology, and Nuclear Medicine named after Professor D.R. Musinov, Semey Medical University, Semey 071400, Kazakhstan; 87madina87@mail.ru (M.A.M.); dana77792@mail.ru (A.M.R.); 2 Office of the Chairman of the Management Board, National Research Oncology Center, Astana 010000, Kazakhstan; zhan11@mail.ru; 3 Center of Endoscopy and Functional Diagnostics, Center of Nuclear Medicine and Oncology, Semey 071400, Kazakhstan; 4 Department of Emergency Medicine, Semey Medical University, Semey 071400, Kazakhstan; semskluda@rambler.ru; 5 Department of Pediatrics, Astana Vision, Astana 010000, Kazakhstan; diana_pak93@bk.ru; 6 Department of Internal Medicine, Semey Medical University, Semey 071400, Kazakhstan; andrey.orekhov@smu.edu.kz; 7 Department of Radionuclide Therapy, Center of Nuclear Medicine and Oncology, Semey 071400, Kazakhstan; azhar597@mail.ru; 8 Center of Cytopathomorphology, Immunohistochemistry, and Translational Oncology, National Research Oncology Center, Astana 010000, Kazakhstan; salta72.72@mail.ru; 9 Department of Tumor and Diagnostic Pathology, Atomic Bomb Disease Institute, Nagasaki University, Nagasaki 852-8523, Japan; moemoe@nagasaki-u.ac.jp

**Keywords:** differentiated thyroid cancer, radioiodine-refractory thyroid cancer, BRAF, TERT promoter, molecular biomarker, Kazakh population, thyroid cancer prognosis

## Abstract

**Background/Objectives:** Radioiodine-refractory differentiated thyroid carcinoma (RAIR-DTC) is associated with limited treatment options and a less favorable clinical course. Combining molecular alterations with clinicopathological characteristics may improve the early identification of patients at risk of radioiodine refractoriness. This study aimed to evaluate the association between molecular genetic alterations, particularly BRAF and TERT promoter mutations, and RAIR-DTC. **Methods:** This retrospective single-center study conducted in Kazakhstan included 167 patients with differentiated thyroid carcinoma treated between 2021 and 2023. Patients were classified into radioiodine-sensitive (*n* = 130) and radioiodine-refractory (*n* = 37) groups. Clinical, histopathological, and molecular genetic characteristics were analyzed. **Results:** Radioiodine refractoriness was observed in all 13 patients (100.0%) with concurrent BRAF and TERT promoter mutations, compared with 24 of 154 patients (15.6%) without this co-mutation (OR = 163.46, 95% CI 9.31–2870.79; *p* < 0.001). The BRAF + TERT co-mutation was also associated with less differentiated histological architecture and a higher T category. In contrast, the BRAF mutation alone showed a substantially weaker association with radioiodine refractoriness, highlighting the incremental value of the combined BRAF + TERT molecular status. **Conclusions:** The concurrent presence of BRAF and TERT promoter mutations, rather than the BRAF mutation alone, may improve the identification of patients at increased risk of radioiodine refractoriness. Integrating molecular profiling with clinicopathological characteristics may support individualized risk stratification and treatment planning.

## 1. Introduction

Thyroid cancer (TC) occupies a leading position among malignant neoplasms of the endocrine system, and its incidence demonstrates a persistent upward trend in many countries worldwide [1,2]. According to global cancer statistics for 2022, thyroid cancer represents one of the major contributors to the overall cancer burden, ranking seventh among all malignancies in the general population and fifth among women. More than 821,000 new cases were reported worldwide, with the incidence rate in women being approximately three times higher than in men. Despite this high detection rate, mortality from thyroid cancer remains relatively low, accounting for approximately 44,000 deaths in 2022, which reflects a marked discrepancy between incidence and lethality [3,4].

Alongside this, the epidemiological profile of thyroid cancer is influenced by various risk factors, including radiation exposure, iodine deficiency, obesity, environmental factors, and the availability of medical diagnostics. The higher prevalence among women may be attributed to the impact of hormonal and reproductive factors, as well as medical and social determinants [5,6]. The observed differences across sex, age, and geographic regions underscore the complex and heterogeneous nature of this disease and highlight the need for further investigation of its clinical, pathological, and molecular characteristics.

Differentiated thyroid carcinoma (DTC), predominantly represented by papillary and follicular histological variants, accounts for more than 90% of all malignant thyroid tumors [7,8]. In most cases, this disease is characterized by a favorable prognosis when standard multimodal treatment is applied, including surgical intervention, radioiodine therapy, and thyroid hormone suppressive therapy [9,10]. However, a subset of patients develops a radioiodine-refractory form of the disease, characterized by a reduced or complete loss of the tumor tissue’s ability to uptake radioactive iodine, as well as a lack of clinically meaningful response to radioiodine therapy. This patient population is associated with a more aggressive disease course, limited therapeutic options, and the need for systemic therapy [11,12].

The pathogenetic basis of radioiodine refractoriness in differentiated thyroid carcinoma is largely associated with reduced thyroid-specific differentiation at the molecular and functional levels, leading to decreased expression of thyroid-specific genes and disruption of iodine metabolism mechanisms. The most significant molecular event is considered to be the loss of functional expression of the sodium-iodide symporter (NIS), accompanied by dysregulation of thyroid peroxidase (TPO), thyroglobulin (TG), and pendrin, which results in reduced ability of tumor cells to accumulate radioactive iodine and, consequently, decreased efficacy of radioiodine therapy [13].

Key intracellular signaling cascades, primarily the MAPK and PI3K/AKT pathways, play a significant role in the development of these alterations by regulating cellular proliferation, differentiation, and survival. Activation of the MAPK pathway, also known as the RAS–RAF–MEK–ERK cascade, is most commonly associated with BRAF and RAS mutations as well as RET/PTC rearrangements, which are regarded as early molecular events in the pathogenesis of differentiated thyroid carcinoma. In contrast, dysregulation of the PI3K/AKT signaling pathway is more frequently observed in aggressive DTC phenotypes and in tumors with reduced thyroid-specific differentiation at the molecular and functional levels, and may be associated with disease progression and loss of responsiveness to radioiodine therapy [14].

According to recent data, the molecular profile of radioiodine-refractory differentiated thyroid carcinoma includes recurrent genetic alterations, primarily BRAF, RAS, and TERT promoter mutations, as well as a range of additional changes associated with tumor progression and aggressive biological behavior [15,16].

It should be noted that individual molecular genetic alterations may be associated not only with the development of radioiodine refractoriness but also with specific clinicopathological characteristics of the tumor. In particular, TERT promoter mutations are associated with a higher likelihood of a radioiodine-refractory course, while the combination of BRAF V600E and TERT promoter mutations is considered an unfavorable molecular pattern associated with a poorer response to radioiodine therapy [17,18].

Notably, the histological pattern of the tumor represents a significant morphological characteristic of differentiated thyroid carcinoma, as it reflects the degree of tumor differentiation and may be associated with the clinical aggressiveness of the neoplasm. Aggressive histological variants, including tall cell, hobnail, columnar cell, diffuse sclerosing, and solid/trabecular patterns, are more frequently associated with an unfavorable disease course, whereas a reduction in the degree of differentiation is accompanied by decreased iodine-uptake capacity of the tumor and, consequently, an increased likelihood of radioiodine refractoriness [19,20]. It should be emphasized that Ki-67 is regarded as an additional marker of proliferative activity in differentiated thyroid carcinoma. Accumulated evidence indicates that an increased Ki-67 index is associated with more aggressive clinicopathological characteristics and an unfavorable disease course, particularly in papillary thyroid carcinoma. Therefore, inclusion of Ki-67 assessment in morphological analysis may contribute to more accurate risk stratification in this patient population [21,22].

Despite significant advances in the study of the molecular biology of thyroid tumors, the relationship between specific molecular genetic alterations and clinicopathological patterns across different groups of radioiodine-refractory thyroid carcinoma remains insufficiently characterized. Clarification of these associations is of fundamental importance both for a deeper understanding of the mechanisms of tumor progression and loss of iodine-uptake function, and for improving risk stratification, prognostic assessment, and the selection of personalized treatment strategies, including the use of targeted and redifferentiation therapies.

## 2. Materials and Methods

### 2.1. Study Design and Patient Population

The study design is a retrospective single-center study of patients with differentiated thyroid carcinoma.

As part of this retrospective study, an analysis was performed of clinical data as well as archived postoperative slides and paraffin-embedded tissue blocks from patients with differentiated thyroid carcinoma who received treatment in the Department of Radionuclide Therapy at the Center of Nuclear Medicine and Oncology in Semey, Republic of Kazakhstan, between January 2021 and December 2023.

### 2.2. Patient Selection

**Figure 1:**
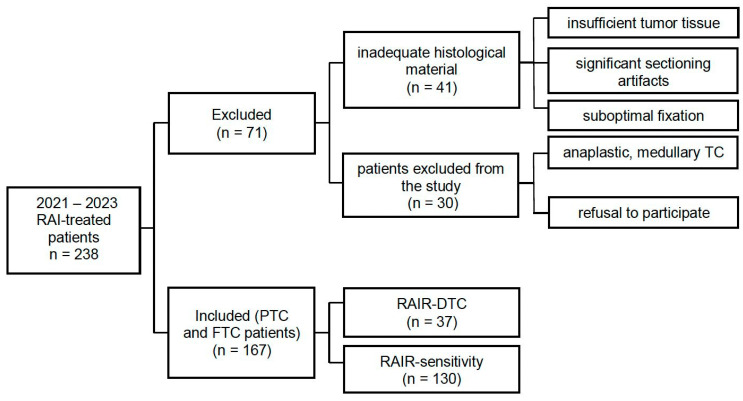
Patient selection scheme for the study.

For the clinical analysis, a patient database was established, initially comprising 238 medical records. In addition, an appropriate number of postoperative histological materials were collected for morphological and molecular analysis. During the study, 71 cases were excluded. The final cohort included 167 patients. Based on the response to radioiodine therapy, patients were divided into two groups: the study group (RAIR-DTC; *n* = 37) and the control group (RAI-sensitive DTC; *n* = 130). The patient selection scheme is presented in Figure 1.

Inclusion criteria: morphologically confirmed differentiated thyroid carcinoma; surgical treatment including total thyroidectomy or total thyroidectomy with modified radical neck dissection (MRND); availability of radioiodine therapy data; availability of clinicopathological data; and availability of Ki-67 and molecular genetic analysis results.

Exclusion criteria: patients with medullary, poorly differentiated or anaplastic thyroid carcinoma; insufficient clinical data; short follow-up period not allowing classification of treatment response; incomplete morphological or molecular data; and histological material unsuitable for analysis due to insufficient tumor tissue volume, presence of significant artifacts during slide preparation, and signs of suboptimal fixation.

All 167 patients included in the final cohort underwent post-therapy whole-body scintigraphy on the fourth day after radioiodine therapy as part of routine treatment assessment. In contrast, 18F-FDG PET/CT was not systematically performed in all patients and was conducted only when clinically indicated, including suspected persistent or recurrent disease, biochemical progression, inconclusive or discordant findings on conventional imaging, or suspicion of the “flip-flop” phenomenon, characterized by reduced or absent radioiodine uptake and increased 18F-FDG uptake in tumor lesions.

Radioiodine-refractory status was assigned retrospectively by the authors of the manuscript through a review of the patients’ medical records, radioiodine treatment history, laboratory findings, post-therapy whole-body scintigraphy, and other available imaging data, including 18F-FDG PET/CT when performed. The same predefined criteria for radioiodine-refractory disease were consistently applied to all patients. RAIR status was not independently assessed or confirmed by a multidisciplinary tumor board.

### 2.3. Radioiodine Therapy Protocol

According to the treatment protocol used in this study, radioiodine therapy was administered to patients with differentiated thyroid carcinoma after total thyroidectomy and after achieving a serum thyroid-stimulating hormone level greater than 30 mIU/L. The administered activity of I-131 was selected according to the treatment objective and the extent of disease. An activity of 3.7 GBq (100 mCi) was used for remnant ablation after surgery in patients without regional or distant metastases or invasion of surrounding tissues. An activity of 5.55 GBq (150 mCi) was administered in the presence of regional lymph node metastases and/or invasion of surrounding tissues. In patients with distant metastases to the lungs and/or bones, activities ranging from 7.4 to 11.1 GBq (200–300 mCi) were used.

When clinically indicated, repeated courses of radioiodine therapy were administered at intervals of at least 6 months, taking into account serum thyroglobulin levels, imaging findings, and the clinical response to previous treatment. Post-therapeutic whole-body scintigraphy was performed on the fourth day after I-131 administration.

The number of radioiodine treatment courses was recorded individually for each patient. Cumulative administered activity (CAA) of I-131 was calculated as the sum of the activities administered during all documented courses of radioiodine therapy. To ensure consistency, all CAA values presented in the Section 3 and the corresponding tables are expressed in megabecquerels (MBq).

### 2.4. Criteria for Radioiodine Refractoriness

Radioiodine refractoriness was defined based on post-therapy whole-body scintigraphy and whole-body positron emission tomography/computed tomography (PET/CT) findings, in accordance with the following criteria for radioiodine refractoriness [23]:

- Presence of one or more foci of well-differentiated thyroid carcinoma visualized on positron emission tomography (PET) but showing no uptake of I-131 on post-therapy whole-body scintigraphy;
- Tumor progression within ≤12 months during radioiodine therapy administered at activities of at least 3.7 GBq, provided that successful ablation of the thyroid remnant had been achieved;
- Absence of tumor lesion regression after a cumulative therapeutic activity of radioactive iodine exceeding 22 GBq (600 mCi).

Additional predictors of radioiodine refractoriness included an increase in thyroglobulin levels and anti-thyroglobulin antibodies without evidence of structural disease progression; significant uptake of 18F-FDG on PET/CT in metastatic lesions; and absence of I-131 uptake on SPECT/CT following administration of therapeutic or diagnostic activity.

Among the 37 patients with RAIR-DTC, 2 patients (5.4%) were classified based on the presence of one or more lesions without therapeutic I-131 uptake, 12 patients (32.4%) based on structural disease progression within 12 months during or after radioiodine therapy, 15 patients (40.5%) based on heterogeneous radioiodine uptake among multiple metastatic lesions, with at least one non-avid lesion, and 8 patients (21.6%) based on absent or progressively decreasing I-131 uptake on sequential post-therapeutic scans. No patient in this subcohort was classified primarily on the basis of absence of lesion regression after a cumulative I-131 activity exceeding 22 GBq (600 mCi). Each patient was counted only once according to the primary classification criterion.

### 2.5. Immunohistochemical, Histopathological and Molecular Analyses

Immunostaining was performed on 4 μm sections of FFPE tissue from DTC cases. A primary antibody against Ki-67 (clone MIB-1; Dako Cytomation Denmark A/S, Glostrup, Denmark) was used. Staining was carried out using the Dako Cytomation Autostainer Universal System (Dako) and the EnVision system (Dako), according to the manufacturer’s instructions.

The Ki-67 proliferation index was independently evaluated by two experienced pathologists. At least 500 tumor cells were counted in areas showing the highest proliferative activity (hot spots) at ×400 magnification. The Ki-67 proliferation index was defined as the percentage of tumor cell nuclei showing positive immunohistochemical staining and was categorized as <5%, 5–10%, or >10%. In cases of discrepant assessments, the slides were jointly reviewed by both pathologists, and a consensus value was recorded.

The histopathological diagnosis was established based on hematoxylin and eosin-stained slides from tissue specimens. All slides were evaluated by two pathologists. DTC subtypes and variants were classified according to the WHO Classification of Endocrine Tumors (5th Edition, 2022). TNM staging was performed according to AJCC 8th edition criteria. Capsular and vascular invasion were recorded quantitatively. Positive surgical margins were assessed based on the presence of tumor cells within inked resection margins. Lymph nodes were evaluated by levels, including the number of examined and metastatic nodes, as well as the presence of extranodal extension.

Molecular analyses were performed at the Department of Tumor and Diagnostic Pathology, Atomic Bomb Disease Institute, Nagasaki University, Japan. Tumor-rich areas were manually macrodissected from 8 µm-thick formalin-fixed, paraffin-embedded (FFPE) tissue sections. Genomic DNA was subsequently extracted using the Maxwell RSC DNA FFPE Kit (Promega, Madison, WI, USA), according to the manufacturer’s instructions. DNA concentration and purity were assessed using a NanoDrop 1000 spectrophotometer (Thermo Fisher Scientific, Wilmington, DE, USA).

The BRAF V600E mutation, TERT promoter mutations at positions C228T and C250T, and NRAS codon 61 mutations were analyzed using droplet digital polymerase chain reaction (ddPCR). The Bio-Rad BRAF V600 Screening Kit (Bio-Rad Laboratories, Inc., Hercules, CA, USA) and NRAS Q61 Screening Kit (Bio-Rad Laboratories, Inc., Hercules, CA, USA) were used for the detection of BRAF and NRAS mutations, respectively, whereas TERT promoter mutations were analyzed using a laboratory-developed ddPCR assay established at the Department of Tumor and Diagnostic Pathology, Atomic Bomb Disease Institute, Nagasaki University, Japan, as previously described [24,25].

Each reaction was performed in a total volume of 20 µL, containing 5 µL of genomic DNA for the BRAF and TERT assays and 1.5 µL for the NRAS assay. Droplet generation, PCR amplification, and fluorescence detection were performed using the Bio-Rad QX200 Droplet Digital PCR System (Bio-Rad Laboratories, Inc., Hercules, CA, USA). The resulting data were analyzed using QuantaSoft software.

Variant allele frequency was calculated as the proportion of mutant droplets among the total number of mutant and wild-type droplets. TERT promoter mutation positivity was defined by the presence of a distinct mutant-positive droplet cluster with a mutant allele fraction above the predefined cutoff and above the background signal observed in wild-type and no-template controls. Based on prior validation of this TERT promoter ddPCR assay [24], the limit of detection was approximately 0.25% mutant allele frequency. Samples with insufficient total TERT promoter DNA copies were considered inadequate for mutation calling. Borderline cases with signals close to the cutoff were not classified as positive unless the mutant signal was clearly above the predefined threshold.

Nuclease-free water was included as a negative control, while DNA samples harboring the corresponding mutations were used as positive controls. Molecular testing was performed independently of the statistical analysis and clinical outcome classification.

### 2.6. Study Variables and Ethical Considerations

Within the study, clinical, clinicopathological, and biological characteristics of patients were analyzed as potential factors associated with radioiodine refractoriness.

Clinical variables included age at diagnosis, sex, TNM stage, body mass index, occupational exposure, presence of chronic thyroid disease and other comorbidities, presence of regional lymph node metastases and distant metastases, cumulative administered activity of radioiodine therapy, and post-therapy SPECT/CT findings.

Clinicopathological characteristics included primary tumor size, histological variant, multifocality, presence of invasion, predominant histological pattern, and TNM stage.

Biological markers included thyroglobulin, anti-thyroglobulin antibodies, Ki-67 proliferation index determined by immunohistochemistry, and molecular genetic alterations, including BRAF V600E mutations, NRAS (codon 61) mutations, and TERT promoter mutations (C228T and C250T).

All variables were considered potential predictors of radioiodine refractoriness and were included in comparative and regression analyses.

The study was approved by the Local Ethics Committee of NCJSC “Semey Medical University” (extract from meeting protocol No. 1b dated 2 November 2023). In addition, prior to hospital admission, all patients provided written informed consent for the anonymized use and dissemination of their medical data. The confidentiality of each participant was ensured in accordance with the ethical code of conduct.

### 2.7. Statistical Analysis

Statistical analyses were performed using IBM SPSS Statistics for Windows, version 20.0. Quantitative variables were assessed for normality using the Shapiro–Wilk test (for sample sizes < 50) or the Kolmogorov–Smirnov test (for sample sizes > 50). In cases of non-normal distribution, quantitative data were described using the median (Me) and interquartile range (Q1–Q3). Categorical variables were presented as absolute numbers and percentages. Comparisons of three or more groups for quantitative variables with non-normal distribution were performed using the Kruskal–Wallis test, with post hoc pairwise comparisons conducted using Dunn’s test with Holm correction. Comparisons of proportions in contingency tables were performed using Pearson’s chi-square test. Post hoc analyses were conducted using Pearson’s chi-square test with Holm correction.

To evaluate the ability of the BRAF V600E/TERT co-mutation to identify patients with radioiodine-refractory differentiated thyroid carcinoma (RAIR-DTC), receiver operating characteristic (ROC) analysis was performed, with calculation of the area under the curve (AUC), 95% confidence intervals (95% CIs), sensitivity, specificity, positive predictive value (PPV), and negative predictive value (NPV). Because all patients harboring the BRAF V600E/TERT co-mutation belonged to the RAIR-DTC group, complete separation of outcomes was observed. To assess the independent association between the BRAF V600E/TERT co-mutation and radioiodine refractoriness, multivariable Firth penalized logistic regression was used. The results are presented as odds ratios (ORs) with 95% confidence intervals. Statistical significance was defined as *p* < 0.05.

## 3. Results

### 3.1. Clinicopathological Characteristics of RAI-Sensitivity and RAIR-DTC Patients

The clinicopathological characteristics of the study groups are summarized in Table 1. Compared with patients with RAI-sensitive DTC, patients with RAIR-DTC showed significant differences in the predominant histological pattern (*p* < 0.001) and in the distributions of the T, N, and M categories (*p* = 0.035, *p* = 0.035, and *p* = 0.028, respectively). The RAIR-DTC group had a higher proportion of T3 tumors (29.7% vs. 14.6%) and N1 disease (45.9% vs. 37.7%). Patients with RAIR-DTC also more frequently underwent total thyroidectomy with modified radical neck dissection (73% vs. 31.5%; *p* < 0.001), received a higher cumulative radioiodine activity (*p* < 0.001), and had a greater lymph node metastatic burden (*p* = 0.004). Capsular invasion was less frequent in the RAIR-DTC group (24.3% vs. 45.4%; *p* = 0.035). No significant between-group differences were observed in age, sex, histological type, tumor size, multifocality, vascular invasion, Ki-67 index, overall disease stage, or distant metastases. Detailed data are presented in Table 1.

The above results highlight clinicopathological factors influencing sensitivity and resistance to radioiodine therapy in patients with differentiated thyroid carcinoma.

Statistical data support the role of histological pattern and tumor stage as significant determinants of refractoriness, particularly in the RAIR-DTC group. These findings may influence treatment selection and support an individualized therapeutic approach in these patients.

### 3.2. Association of Molecular Alterations with Clinicopathological Features and Radioiodine-Refractory Phenotype in Differentiated Thyroid Carcinoma

Comparison of clinicopathological characteristics between molecular subgroups (wild-type, BRAF-only, and BRAF + TERT) demonstrated (Table 2) that the combined BRAFV600E + TERT molecular profile is associated with the most unfavorable tumor phenotype. Statistically significant differences between the groups were observed in the frequency of radioiodine refractoriness, predominant histological pattern, and T category. Importantly, all tumors with the combined BRAFV600E + TERT mutation were radioiodine-refractory (100%), whereas in the wild-type and BRAF-only groups, radioiodine refractoriness was observed in only 14.7% and 15.8% of patients, respectively (*p* < 0.001). These findings indicate a strong association between the double mutation and the development of a radioiodine-refractory phenotype.

Age, sex, histological type, tumor size, multifocality, disease stage, N and M categories, extent of surgical treatment, and cumulative radioiodine activity did not differ significantly between the groups, suggesting that the observed differences are less dependent on baseline clinical characteristics and are more strongly associated with the molecular status of the tumor. At the same time, complete encapsulation was observed significantly less frequently in the BRAFV600E + TERT subgroup (23.1%) compared with the wild-type (61.8%) and BRAF-only (55.8%) groups, reaching borderline statistical significance (*p* = 0.050), which further suggests a tendency toward more invasive growth in the presence of the double mutation.

**Figure 2:**
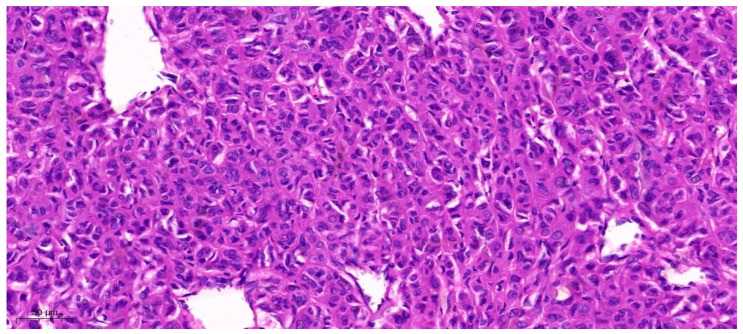
Representative histological image of differentiated thyroid carcinoma with a predominantly solid growth pattern. The tumor is composed of solid nests and trabecular structures of neoplastic follicular cells separated by delicate fibrovascular septa. This architectural pattern may be observed in both papillary and follicular thyroid carcinomas. Hematoxylin and eosin staining. Original magnification ×400; scale bar, 50 µm.

**Figure 3:**
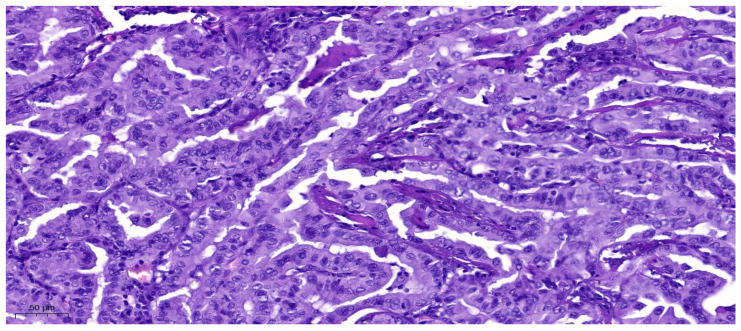
Representative hematoxylin and eosin-stained image of papillary thyroid carcinoma with a trabecular growth pattern. The tumor contains solid fields and trabeculae composed of atypical polygonal and oval thyroid cells with moderate nuclear atypia, hyperchromatic nuclei, and focally irregular nuclear contours. The image represents a BRAF V600E/TERT promoter co-mutated tumor from a patient with RAIR-DTC. Original magnification, ×400; scale bar, 50 µm.

**Figure 4:**
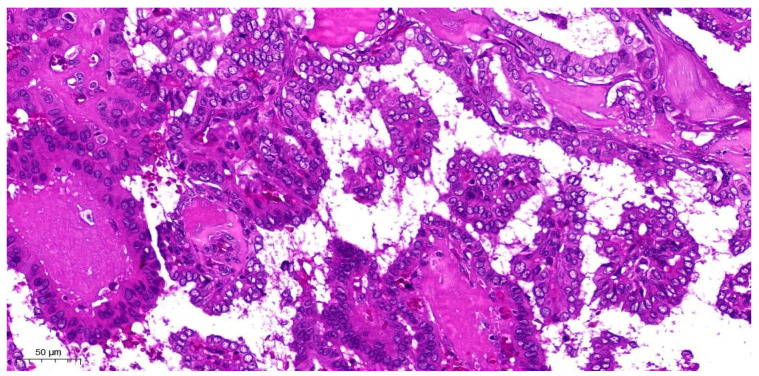
Representative hematoxylin and eosin-stained image of papillary thyroid carcinoma with a conventional papillary growth pattern. The papillary structures are lined by a single layer of atypical thyrocytes with enlarged hyperchromatic nuclei, irregular nuclear contours, and prominent nuclear grooves. The image represents a BRAF V600E/TERT promoter co-mutated tumor from a patient with RAIR-DTC. Original magnification, ×400; scale bar, 50 µm.

Significant differences were also identified with respect to predominant histological pattern (*p* < 0.001). The BRAFV600E + TERT subgroup demonstrated a shift in morphological spectrum toward more aggressive architectural variants, including trabecular and less differentiated components, whereas the wild-type and BRAF-only groups more frequently retained typical well-differentiated patterns (Figure 2, Figure 3 and Figure 4). This distribution supports the notion that the combined mutation is associated not only with molecular alterations per se but also with tumor morphological remodeling toward a biologically more aggressive phenotype.

The Ki-67 proliferation index demonstrated a borderline trend toward differences (*p*= 0.055), with the BRAFV600E + TERT group more frequently showing moderate proliferative activity, which may further reflect a more active biological behavior of the tumor. The T category also differed significantly between subgroups (*p* = 0.002), confirming the association between molecular profile and local tumor extent.

Taken together, these findings indicate that the combined BRAFV600E + TERT status, rather than isolated BRAFV600E mutation, is associated with the most aggressive clinico-pathological features, including radioiodine refractoriness and less favorable morphological architecture. This supports the consideration of the double mutation as a potential marker of adverse disease course and as a more informative molecular indicator of the radioiodine-refractory phenotype compared with isolated BRAF status.

### 3.3. Association of BRAF + TERT Co-Mutation with Clinicopathological Characteristics and Radioiodine-Refractory Phenotype in Differentiated Thyroid Carcinoma

When comparing tumors with the BRAF + TERT co-mutation (*n* = 13) and cases without this combination (*n* = 154), it was found that the presence of the double mutation is strongly associated with radioiodine refractoriness: all BRAF + TERT cases were radioiodine-refractory, whereas in the comparison group, radioiodine refractoriness was observed in only 15.6% of patients (*p* < 0.001).

Age, sex, histological type, tumor size, multifocality, Ki-67 index, N and M categories, and disease stage did not differ significantly between the groups. However, tumors with the BRAF + TERT co-mutation less frequently demonstrated complete encapsulation (23.1% vs. 57.1%; *p* = 0.022), and exhibited significant alterations in the predominant histological pattern toward less differentiated, solid and trabecular components (*p* < 0.001). They were also more frequently classified as T3 tumors (*p* = 0.029). In addition, patients with BRAF + TERT underwent total thyroidectomy with modified radical neck dissection (MRND) more often (*p* = 0.039).

The BRAF V600E/TERT co-mutation was detected in 13 patients (7.8%). All tumors harboring this co-mutation belonged to the RAIR-DTC group, whereas no cases of the BRAF + TERT co-mutation were identified among patients with RAI-sensitive DTC. Fisher’s exact test demonstrated a highly significant association between the BRAF + TERT co-mutation and tumor radioiodine refractoriness (*p* < 0.001). Because of complete separation of outcomes, standard logistic regression was not informative.

**Figure 5:**
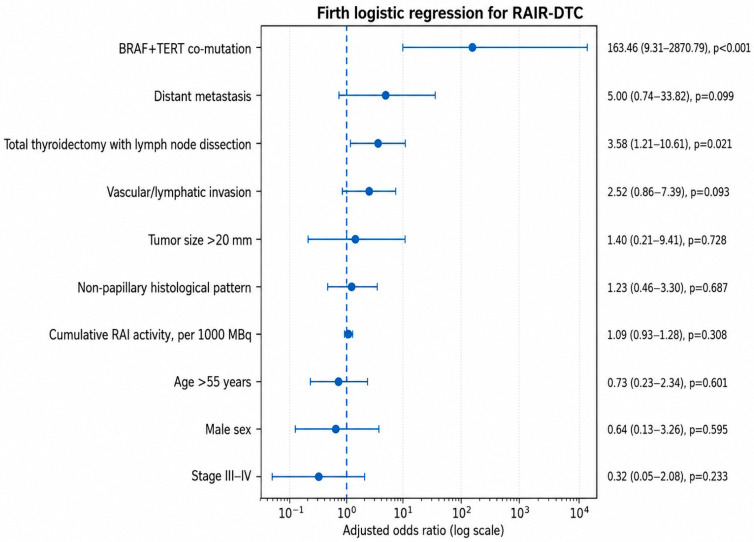
Forest plot of adjusted odds ratios for factors associated with radioiodine-refractory differentiated thyroid carcinoma. Dots represent adjusted odds ratios, horizontal lines indicate 95% confidence intervals, and the vertical dashed line indicates the null value (OR = 1).

To assess the independent association between the BRAF V600E/TERT promoter co-mutation and radioiodine refractoriness, multivariable Firth penalized logistic regression was performed, which provides valid estimates in the presence of complete separation (Figure 5).

The model included age, sex, tumor size, vascular/lymphatic invasion, histological tumor subtype, extent of surgery, cumulative administered radioiodine activity, presence of distant metastases, and disease stage. After adjustment for these clinicopathological factors, the BRAF V600E/TERT co-mutation remained strongly and independently associated with RAIR-DTC. The presence of the co-mutation increased the odds of RAIR-DTC more than 160-fold (OR = 163.46, 95% CI 9.31–2870.79; *p* < 0.001). The wide confidence interval reflects the limited number of patients with the co-mutation (*n* = 13) and the fact that all co-mutation cases were identified exclusively in the RAIR-DTC group; therefore, this result should be interpreted with caution.

Overall, these findings indicate an association of the BRAF + TERT co-mutation with a more aggressive clinicopathological and radioiodine-refractory phenotype of differentiated thyroid carcinoma (Table 3).

**Figure 6:**
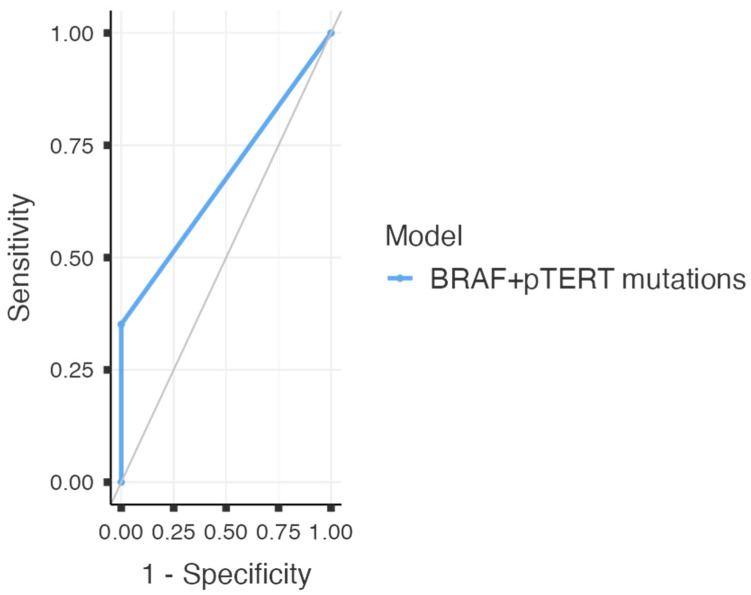
Receiver operating characteristic (ROC) analysis of the predictive ability of the BRAF V600E/TERT promoter co-mutation for identifying RAIR-DTC.

To assess the ability of the BRAF V600E/TERT promoter co-mutation to identify patients with RAIR-DTC, receiver operating characteristic (ROC) analysis was performed (Figure 6). The ROC analysis showed that the BRAF V600E/TERT promoter co-mutation had moderate discriminatory ability for distinguishing patients with RAIR-DTC from those with RAI-sensitive differentiated thyroid carcinoma (AUC = 0.676, 95% CI 0.598–0.754; *p* < 0.001). The co-mutation demonstrated a sensitivity of 35.1% and a specificity of 100.0%. The positive predictive value (PPV) was 100.0%, whereas the negative predictive value (NPV) was 84.4%.

## 4. Discussion

The present study is based on a histologically and molecularly characterized subcohort of 167 patients who were included in the larger cohort of 373 patients reported in our previous publication [26]. That study described clinicopathological factors associated with radioiodine refractoriness, the overall distribution of BRAF V600E, NRAS, and TERT promoter mutations, and the exclusive occurrence of the BRAF V600E/TERT promoter co-mutation in patients with RAIR-DTC.

Although the present cohort is entirely contained within the previously published cohort, the current manuscript addresses distinct analytical objectives. Specifically, it presents detailed morphomolecular and clinicopathological associations, comparisons between BRAF/TERT-defined molecular subgroups, multivariable Firth penalized logistic regression, and ROC analysis, none of which were included in the previous publication.

In the analyses newly presented in this manuscript, the BRAF V600E/TERT promoter co-mutation was associated with a more aggressive clinicopathological profile, including radioiodine refractoriness, solid and trabecular architectural components, and a more advanced T category. In contrast, isolated BRAF V600E mutation did not demonstrate associations of comparable magnitude. These findings suggest that co-mutation status may provide additional discriminatory and clinicopathological information beyond that provided by BRAF V600E mutation alone.

Our findings are consistent with current concepts regarding the biology of radioiodine-refractory thyroid carcinoma. It is well established that activation of the MAPK signaling pathway via the BRAF V600E mutation leads to suppression of thyroid differentiation gene expression, including NIS, TPO, and TG, which results in reduced iodine-uptake capacity of tumor cells and decreased therapeutic efficacy. However, the BRAF mutation alone is also observed in clinically indolent forms of papillary thyroid carcinoma, which limits its independent prognostic value. This is consistent with our data, where the BRAF-only group was, in most parameters, closer to the wild-type group than to the double-mutation group.

In contrast, TERT promoter mutations are considered a marker of tumor progression and an unfavorable prognosis. It has previously been shown that the presence of TERT promoter mutations is associated with higher rates of recurrence, metastasis, and reduced survival in patients with differentiated thyroid carcinoma [27]. Particularly important is the synergism between BRAF and TERT, whereby MAPK pathway activation enhances transcriptional activity of the mutant TERT promoter, contributing to more aggressive tumor growth. This likely explains the finding that, in our cohort, all cases with the BRAF + TERT co-mutation were radioiodine-refractory.

Our results are also consistent with the findings of Yang et al., who demonstrated that TERT promoter mutations represent an independent predictor of a radioiodine-refractory course in patients with metastatic differentiated thyroid carcinoma, while their combination with BRAF further enhances this effect [28]. More recent studies have confirmed that patients with the BRAF + TERT co-mutation are characterized by worse clinical outcomes, shorter time to disease progression, and a higher probability of loss of tumor radioiodine-uptake function [29].

One of the most significant observations of our study is the association of the BRAF + TERT co-mutation with alterations in the histological architectural pattern of differentiated thyroid carcinoma. In this subgroup, solid, trabecular, and mixed architectural components were more frequently observed, whereas more conventional well-differentiated DTC patterns predominated in the remaining groups. These findings may indicate that solid and trabecular architectural components within DTC represent an intermediate morphological phenotype linking molecular alterations with the development of clinical radioiodine refractoriness. In the study by Hong CM and Ahn BC [30], it is emphasized that the ability of a tumor to concentrate radioiodine is considered a feature of a more differentiated phenotype, whereas loss of iodine uptake and increased FDG metabolism are characteristic of a less differentiated and biologically more aggressive tumor state [31]. It has previously been reported that tumors that have lost the ability to uptake radioactive iodine often demonstrate a less differentiated architecture, increased cellular atypia, and enhanced glycolytic metabolism [32,33].

An additional important finding is the observed association of the co-mutation with a higher T category, indicating a link between this molecular profile and locally aggressive tumor growth. Although differences in N and M categories did not reach statistical significance, this is likely attributable to the small number of patients in the BRAF + TERT group (*n* = 13), which limits the statistical power of the analysis.

From a clinical perspective, these findings have important practical implications. Determination of BRAF + TERT status at the preoperative or early postoperative stage may potentially be used to identify high-risk patients with a substantially increased likelihood of failure of standard radioiodine therapy. In such patients, more intensive monitoring, early application of functional imaging modalities, and consideration of personalized treatment strategies, including targeted therapy or redifferentiation approaches, may be justified.

### Limitations

This study has several limitations that should be considered when interpreting the results. First, the study design was retrospective, which may have limited the completeness of clinical data and does not exclude the potential influence of unaccounted confounding factors on the observed results.

Despite an adequate overall sample size, the number of patients in the BRAF + TERT co-mutation subgroup was relatively small, which may have reduced the statistical power of the analysis in assessing rare clinical events, including specific patterns of metastatic spread and less common histological variants. In addition, molecular analysis was restricted to patients for whom adequate FFPE tumor material and complete molecular data were available. Therefore, the analyzed cohort may not fully represent the entire eligible DTC population, and selection bias related to tissue and data availability cannot be excluded. It should also be noted that radioiodine refractoriness was defined based on the clinical course of the disease and follow-up data; therefore, the observed associations reflect the relationship between molecular status and an already established phenotype, but do not allow definitive conclusions regarding causality.

In addition, this was a single-center study conducted at a specialized nuclear medicine and oncology center. Consequently, the study population may have included a disproportionate number of patients with more complex, advanced, or high-risk disease who were referred for radioiodine treatment. This referral pattern may have introduced selection bias and may limit the generalizability of the findings to the broader population of patients with differentiated thyroid carcinoma in Kazakhstan.

Furthermore, survival outcomes, including recurrence-free, disease-free, progression-free, and overall survival, were not evaluated. Therefore, the present findings do not allow direct conclusions regarding the association of the BRAF + TERT co-mutation with long-term prognosis or survival and should be interpreted as evidence of its association with radioiodine refractoriness and the clinicopathological characteristics assessed in this study.

One limitation of this study is the absence of standardized data on the individual duration of follow-up. Therefore, the median follow-up period could not be calculated, and the later development of radioiodine refractoriness cannot be completely excluded in some patients classified as having radioiodine-sensitive DTC.

Finally, although a unified national database with standardized diagnostic and treatment approaches was used, the findings require external validation in independent multicenter cohorts and across different patient populations.

## 5. Conclusions

The obtained results indicate that radioiodine refractoriness in differentiated thyroid carcinoma is associated not only with individual clinicopathological characteristics but also with the tumor’s molecular profile. In the present cohort, the BRAF V600E/TERT promoter co-mutation showed the strongest association with the radioiodine-refractory phenotype and was associated with solid and trabecular architectural components and a higher T category.

In contrast, the isolated BRAF mutation did not demonstrate comparable prognostic significance, underscoring the importance of assessing combined molecular alterations. Thus, the BRAF + TERT co-mutation may be considered a potential marker of an aggressive and radioiodine-refractory phenotype of differentiated thyroid carcinoma.

However, because of the retrospective single-center design, the limited number of co-mutated tumors, and the absence of an independent validation cohort, the present findings do not establish the co-mutation as a validated predictive biomarker or as a basis for treatment selection. Further prospective multicenter studies are required to confirm the prognostic significance of the observed associations.

## Figures and Tables

**Table 1:** Clinicopathological characteristics of RAI-sensitivity and RAIR-DTC patients.

Parameter	All Patients, *n* = 167	Study Groups		*p*
		RAI-Sensitive DTC, *n* = 130 (77.8%)	RAIR-DTC *n* = 37 (22.2%)	
Age (Median), years	49.2 (15)	49 (15.7)	49.3 (14.8)	0.926
Age, *n* (%):				0.694
<55 years	106 (63.5)	81 (62.3)	25 (67.6)	
>55 years	61 (36.5)	49 (37.7)	12 (32.4)	
Sex, *n* (%):				0.616
female	142 (85)	112 (86.2)	30 (81.1)	
male	25 (15)	18 (13.8)	7 (18.9)	
Histology:				1
Papillary carcinoma	159 (95.2)	124 (95.4)	35 (94.6)	
Follicular carcinoma	8 (4.8)	6 (4.6)	2 (5.4)	
Tumor size (mm)				0.682
≤10	40 (24)	33 (25.4)	7 (18.9)	
>10	127 (76)	97 (74.6)	30 (81.1)	
Multifocality, *n* (%)	52 (31.1)	39 (30)	13 (35.1)	0.694
Complete encapsulation, *n* (%)	91 (54.5)	76 (58.5)	15 (40.5)	0.081
Capsular invasion, *n* (%)	68 (40.7)	59 (45.4)	9 (24.3)	0.035
Vascular invasion, *n* (%)	22 (13.2)	19 (14.6)	3 (8.1)	0.413
Predominant histologic pattern:				<0.001
Papillary	77 (46.1)	62 (47.7)	15 (40.5)	
Follicular	0	0	0	
P > F	74 (44.3)	61 (46.9)	13 (35.1)	
F > P	6 (3.6)	5 (3.8)	1 (2.7)	
Papillary-trabecular pattern	4 (2.4)	0 (0.0)	4 (10.8)	
Less differentiated architectural pattern in DTC	1 (0.6)	0 (0.0)	1 (2.7)	
P > F with trabecular component 5%	5 (3)	2 (1.5)	3 (8.1)	
Ki 67, %:				0.221
<5%	76 (45.5)	55 (42.3)	21 (56.8)	
5–10%	60 (35.9)	48 (36.9)	12 (32.4)	
>10%	31 (18.6)	27 (20.8)	4 (10.8)	
T-category				0.035
T1	83 (49.7%)	63 (48.5%)	20 (54.1%)	
T2	39 (23.4%)	36 (27.7%)	3 (8.1%)	
T3	30 (18%)	19 (14.6%)	11 (29.7%)	
T4	15 (9%)	12 (9.2%)	3 (8.1%)	
N-category				
0	98 (58.7%)	80 (61.5%)	18 (48.6%)	0.035
1	66 (39.5%)	49 (37.7%)	17 (45.9%)	
M-category				0.028
M0	160 (95.8%)	126.0 (96.9%)	34 (91.9%)	
M1	5 (3%)	4 (3.1%)	1 (2.7%)	
Mx	2 (1.2%)	0 (0%)	2 (5.4%)	
Disease stage				0.362
Stage I	104 (62.3%)	79 (60.8%)	25 (67.6%)	
Stage II	33 (19.8%)	25 (19.2%)	8 (21.6%)	
Stage III	20 (12.0%)	16 (12.3%)	4 (10.8%)	
Stage IV	10 (6.0%)	10 (7.7%)	0 (0%)	
Extent of surgery				<0.001
Total thyroidectomy	99 (59.3)	89 (68.5)	10 (27)	
Total thyroidectomy with modified radical neck dissection (MRND)	68 (40.7)	41 (31.5)	27 (73)	
CAA (Cumulative administered activity) (MBq); mean (SD)	5841.2 (3065.4)	5406.9 (2514.1)	7454.3 (4236.1)	<0.001
Lymph node metastasis, *n* (%) median (IQR)	0 (2)	0 (1)	1 (5)	0.004
Distant metastasis, *n* (%)	9 (5.4)	5 (3.8)	4 (10.8)	0.214

**Table 2:** Association of molecular alterations (BRAF and BRAF + TERT) with clinicopathological features and radioiodine-refractory phenotype in differentiated thyroid carcinoma.

Variable	Wild-Type (*n* = 34)	BRAF Only (*n* = 120)	BRAFV600E + TERT (*n* = 13)	*p*-Value
Radioiodine refractoriness	5 (14.7)	19 (15.8)	13 (100)	<0.001
Age, Me (Q1–Q3)	48.50 (33–63.75)	47 (39–59)	51 (44–64)	0.652
Female sex, *n* (%):	29 (85.3)	103 (85.8)	10 (76.9)	0.693
Histology:				0.374
Papillary carcinoma	31 (91.2)	115 (95.8)	13 (100)	
Follicular carcinoma	3 (8.8)	5 (4.2)	0	
Tumor size, mm				0.728
≤10	6 (17.6)	31 (25.8)	3 (23.1)	
10–20	27 (79.4)	83 (69.2)	10 (76.9)	
>20	1 (2.9)	6 (5)	0 (0)	
Multifocality, *n* (%)	11 (32.4)	36 (30)	5 (38.5)	0.81
Complete encapsulation, *n* (%)	21 (61.8)	67 (55.8)	3 (23.1)	0.05
Capsular invasion, *n* (%)	15 (44.1)	48 (40)	5 (38.5)	0.898
Vascular invasion, *n* (%)	9 (26.4)	13 (10.8)	0	0.021
Predominant histologic pattern:				<0.001
Papillary	77 (46.1)	62 (47.7)	15 (40.5)	
Follicular	0	0	0	
P > F	74 (44.3)	61 (46.9)	13 (35.1)	
F > P	6 (3.6)	5 (3.8)	1 (2.7)	
Papillary-trabecular pattern	4 (2.4)	0 (0.0)	4 (10.8)	
Less differentiated architectural pattern in DTC	1 (0.6)	0 (0.0)	1 (2.7)	
P > F with trabecular component 5%	5 (3)	2 (1.5)	3 (8.1)	
Ki 67, %:				0.055
<5%	19 (55.9)	53 (44.2)	4 (30.8)	
5–10%	6 (17.6)	46 (38.3)	8 (61.5)	
>10%	9 (26.5)	21 (17.5)	1 (7.7)	
Extent of surgery:				0.086
Total thyroidectomy	20 (58.8)	75 (62.5)	4 (30.8)	
Total thyroidectomy with modified radical neck dissection (MRND)	14 (41.2)	45 (37.5)	9 (69.2)	
CAA (Cumulative administered activity) (MBq), mean (SD)	5658.4 (2707.0)	5808.3 (3171.4)	6660.6 (2980.8)	0.614
Disease stage				0.119
Stage I	18 (52.9)	80 (66.7)	6 (46.2)	
Stage II	8 (23.5)	21 (17.5)	4 (30.8)	
Stage III	3 (8.8)	14 (11.7)	3 (23.1)	
Stage IV	5 (14.7)	5 (4.2)	0 (0)	
T-category				0.002 *p* Wild type–BRAF = 0.026 *p* Wild type–BRAF + TERT = 0.048 *p* BRAF–BRAF + TERT = 0.048
T1	11 (32.4)	66 (55)	6 (46.2)	
T2	10 (29.4)	28 (23.3)	1 (7.7)	
T3	5 (14.7)	19 (15.8)	6 (46.2)	
T4	8 (23.5)	7 (5.8)	0 (0)	
N-category				0.878
0	23 (67.6)	68 (56.7)	7 (53.8)	
1	11 (32.4)	49 (40.8)	6 (46.2)	
M-category				0.69
M0	32 (94.1)	115 (95.8)	13 (100)	
M1	2 (5.9)	3 (2.5)	0 (0)	
Mx	0 (0)	2 (1.7)	0 (0)	

*Kruskal–Wallis test* *; Pearson’s chi-square test.*

**Table 3:** Association of BRAF + TERT co-mutation with clinicopathological characteristics in differentiated thyroid carcinoma.

Parameter	Molecular Subgroup		*p*
	BRAF + TERT, *n* = 13	Non-BRAF + TERT, *n* = 154	
Radioiodine refractoriness	13 (100)	24 (15.6)	<0.001 b
Age (Me, Q1–Q3), years	51 (44–64)	47.5 (37–62.75)	0.359 a
Sex:			
Female	10 (76.9)	132 (85.7)	0.416 b
Histology:			1 a
Papillary carcinoma	13 (100)	146 (94.8)	
Follicular carcinoma	-	8 (5.2)	
Tumor size (mm)			0.724 c
≤10	3 (23.1)	37 (24)	
10–20	10 (76.9)	110 (71.4)	
>20	0 (0)	7 (4.5)	
Multifocality, *n* (%)	5 (38.5)	47 (30.5)	0.546 b
Complete encapsulation, *n* (%)	3 (23.1)	88 (57.1)	0.022 b
Capsular invasion, *n* (%)	5 (38.5)	63 (40.9)	1 b
Vascular invasion, *n* (%)	0	22 (14.3)	0.221 b
Predominant histologic pattern:			
Papillary	4 (30.8)	73 (47.4)	<0.001c
Follicular	0	0	
P > F	4 (30.8)	70 (45.5)	
F > P	0 (0)	6 (3.9)	
Papillary-trabecular pattern	1 (7.7)	3 (1.9)	
Less differentiated architectural pattern in DTC	1 (7.7)	0 (0)	
P>F with trabecular component 5%	3 (23.1)	2 (1.3)	
Ki 67, %:			0.126 c
<5%	4 (30.8)	72 (46.8)	
5–10%	8 (61.5)	52 (33.8)	
>10%	1 (7.7)	30 (19.5)	
T-category			0.029
T1	6 (46.2)	77 (50)	
T2	1 (7.7)	38 (24.7)	
T3	6 (46.2)	24 (15.6)	
T4	0 (0)	15 (9.7)	
N-category			0.926
0	7 (53.8)	91 (59.1)	
1	6 (46.2)	60 (39)	
M-category			0.735
M0	13 (100)	147 (95.5)	
M1	0 (0)	5 (3.2)	
Mx	0 (0)	2 (1.3)	
Disease stage			0.290 c
Stage I	6 (46.2)	98 (63.6)	
Stage II	4 (30.8)	29 (18.8)	
Stage III	3 (23.1)	17 (11)	
Stage IV	0 (0)	10 (6.5)	
Extent of surgery:			
Total thyroidectomy	4 (30.8)	95 (61.7)	0.039
Total thyroidectomy with modified radical neck dissection (MRND)	9 (69.2)	59 (38.3)	

*a—Mann–Whitney U test; b—Fisher’s exact test; c—Pearson’s chi-square test.*

## Data Availability

The data presented in this study are available on request from the corresponding author. The data are not publicly available due to patient privacy and ethical restrictions.

## Abbreviations

The following abbreviations are used in this manuscript:

TC	Thyroid cancer
DTC	Differentiated thyroid carcinoma
RAIR-DTC	Radioiodine-refractory differentiated thyroid carcinoma
RAI-sensitive DTC	Radioiodine-sensitive differentiated thyroid carcinoma
RAI	Radioactive iodine/radioiodine
NIS	Sodium-iodide symporter
TPO	Thyroid peroxidase
TG	Thyroglobulin
MAPK	Mitogen-activated protein kinase pathway
PI3K/AKT	Phosphoinositide 3-kinase/protein kinase B pathway
RAS-RAF-MEK-ERK	RAS-RAF-MEK-ERK signaling cascade
BRAF	B-Raf proto-oncogene
BRAF V600E	BRAF V600E mutation
RAS	RAS proto-oncogene family
NRAS	Neuroblastoma RAS viral oncogene homolog
RET/PTC	Rearranged during transfection/papillary thyroid carcinoma rearrangement
TERT	Telomerase reverse transcriptase
TERT-p	TERT promoter mutation
DNA	Deoxyribonucleic acid
Ki-67	Ki-67 proliferation index
PI	Proliferation index
18F-FDG	18F-fluorodeoxyglucose
PET/CT	Positron emission tomography/computed tomography
SPECT/CT	Single-photon emission computed tomography/computed tomography
GBq	Gigabecquerel
mCi	Millicurie
FFPE	Formalin-fixed paraffin-embedded
WHO	World Health Organization
TNM	Tumor, Node, Metastasis classification
AJCC 8	American Joint Committee on Cancer, 8th edition
TT	Total thyroidectomy
MRND	Modified radical neck dissection
TT + MRND	Total thyroidectomy with modified radical neck dissection
n	Number of cases

## References

[1] Pizzato M., Li M., Vignat J., Laversanne M., Singh D., La Vecchia C., Vaccarella S. (2022). The epidemiological landscape of thyroid cancer worldwide: GLOBOCAN estimates for incidence and mortality rates in 2020. Lancet Diabetes Endocrinol..

[2] Lyu Z., Zhang Y., Sheng C., Huang Y., Zhang Q., Chen K. (2024). Global burden of thyroid cancer in 2022: Incidence and mortality estimates from GLOBOCAN. Chin. Med. J..

[3] Bray F., Laversanne M., Sung H., Ferlay J., Siegel R.L., Soerjomataram I., Jemal A. (2024). Global cancer statistics 2022: GLOBOCAN estimates of incidence and mortality worldwide for 36 cancers in 185 countries. CA Cancer J. Clin..

[4] Miranda-Filho A., Lortet-Tieulent J., Bray F., Cao B., Franceschi S., Vaccarella S., Dal Maso L. (2021). Thyroid cancer incidence trends by histology in 25 countries: A population-based study. Lancet Diabetes Endocrinol..

[5] Togawa K., Ahn H.S., Auvinen A., Bauer A.J., Brito J.P., Davies L., Kesminiene A., Laurier D., Ostroumova E., Pacini F. (2018). Long-term strategies for thyroid health monitoring after nuclear accidents: Recommendations from an Expert Group convened by IARC. Lancet Oncol..

[6] Zane M., Parello C., Pennelli G., Townsend D.M., Merigliano S., Boscaro M., Toniato A., Baggio G., Pelizzo M.R., Rubello D. (2017). Estrogen and thyroid cancer is a stem affair: A preliminary study. Biomed. Pharmacother..

[7] Fagin J.A., Wells S.A. (2016). Biologic and clinical perspectives on thyroid cancer. N. Engl. J. Med..

[8] Haugen B.R., Alexander E.K., Bible K.C., Doherty G.M., Mandel S.J., Nikiforov Y.E., Pacini F., Randolph G.W., Sawka A.M., Schlumberger M. (2016). 2015 American Thyroid Association management guidelines for adult patients with thyroid nodules and differentiated thyroid cancer. Thyroid.

[9] Jin Y., Van Nostrand D., Cheng L., Liu M., Chen L. (2018). Radioiodine refractory differentiated thyroid cancer. Crit. Rev. Oncol. Hematol..

[10] Liu Y., Wang J., Hu X., Pan Z., Xu T., Xu J., Jiang L., Huang P., Zhang Y., Ge M. (2023). Radioiodine therapy in advanced differentiated thyroid cancer: Resistance and overcoming strategy. Drug Resist. Updates.

[11] Van Nostrand D. (2018). Radioiodine refractory differentiated thyroid cancer: Time to update the classifications. Thyroid.

[12] Volpe F., Nappi C., Zampella E., Di Donna E., Maurea S., Cuocolo A., Klain M. (2024). Current advances in radioactive iodine-refractory differentiated thyroid cancer. Curr. Oncol..

[13] Shen H., Zhu R., Liu Y., Hong Y., Ge J., Xuan J., Niu W., Yu X., Qin J.J., Li Q. (2024). Radioiodine-refractory differentiated thyroid cancer: Molecular mechanisms and therapeutic strategies for radioiodine resistance. Drug Resist. Updates.

[14] Chung J.H. (2020). BRAF and TERT promoter mutations: Clinical application in thyroid cancer. Endocr. J..

[15] Yuan X., Yuan H., Zhang N., Liu T., Xu D. (2022). Thyroid carcinoma-featured telomerase activation and telomere maintenance: Biology and translational/clinical significance. Clin. Transl. Med..

[16] Cao J., Zhu X., Sun Y., Li X., Yun C., Zhang W. (2022). The genetic duet of BRAF V600E and TERT promoter mutations predicts the poor curative effect of radioiodine therapy in papillary thyroid cancer. Eur. J. Nucl. Med. Mol. Imaging.

[17] Vuong H.G., Altibi A.M.A., Duong U.N.P., Hassell L. (2017). Prognostic implication of BRAF and TERT promoter mutation combination in papillary thyroid carcinoma: A meta-analysis. Clin. Endocrinol..

[18] van Gerwen M., Cerutti J.M., Mendes T.B., Brody R., Genden E., Riggins G.J., Taioli E. (2023). TERT and BRAF V600E mutations in thyroid cancer of World Trade Center responders. Carcinogenesis.

[19] Nilsson J.N., Siikanen J., Condello V., Jatta K., Saini R., Hedman C., Ihre Lundgren C., Juhlin C.C. (2023). Iodine avidity in papillary and poorly differentiated thyroid cancer is predicted by immunohistochemical and molecular work-up. Eur. Thyroid J..

[20] Coca-Pelaz A., Shah J.P., Hernandez-Prera J.C., Ghossein R.A., Rodrigo J.P., Hartl D.M., Olsen K.D., Shaha A.R., Zafereo M., Suarez C. (2020). Papillary thyroid cancer-aggressive variants and impact on management: A narrative review. Adv. Ther..

[21] Peștean C., Pavel A., Piciu D. (2024). Clinical and paraclinical considerations regarding Ki-67’s role in the management of differentiated thyroid carcinoma: A literature review. Medicina.

[22] Lindfors H., Karlsen M., Karlton E., Zedenius J., Larsson C., Lundgren C.I., Juhlin C.C., Shabo I. (2023). Thyroglobulin expression, Ki-67 index, and lymph node ratio in the prognostic assessment of papillary thyroid cancer. Sci. Rep..

[23] Boudina M. (2023). Radioiodine refractory differentiated thyroid cancer. Hell. J. Nucl. Med..

[24] Tanaka A., Matsuse M., Saenko V., Nakao T., Yamanouchi K., Sakimura C., Yano H., Nishihara E., Hirokawa M., Suzuki K. (2019). TERTmRNA Expression as a Novel Prognostic Marker in Papillary Thyroid Carcinomas. Thyroid.

[25] Nakao T., Matsuse M., Saenko V., Rogounovitch T., Tanaka A., Suzuki K., Higuchi M., Sasai H., Sano T., Hirokawa M. (2021). Preoperative detection of the TERT promoter mutations in papillary thyroid carcinomas. Clin. Endocrinol..

[26] Rakhmankulova A., Pak L., Pivina L., Burkitbayev Z., Orekhov A., Pak D., Bolsynbekova S., Pivin M., Seitkhanova D., Kudaiberdinov K. (2026). Association between clinical and pathological factors and risk of radioiodine refractory in patients with differentiated thyroid carcinoma. Front. Endocrinol..

[27] Nhung L.T.T., Hoan N.X., Giang D.P., Dung D.T., Phuong N.T., Hanh N.T.M., Khanh L.V., Hang N.T., Ha L.N., Tong H.V. (2025). Prognostic significance of BRAF V600E and TERT promoter mutations in radioiodine resistance and recurrence of differentiated thyroid cancer. Medicine.

[28] Yang X., Li J., Li X., Liang Z., Gao W., Liang J., Cheng S., Lin Y. (2017). TERT promoter mutation predicts radioiodine-refractory character in distant metastatic differentiated thyroid cancer. J. Nucl. Med..

[29] Tan G., Jin B., Qian X., Wang Y., Zhang G., Agyekum E.A., Wang F., Shi L., Zhang Y., Mao Z. (2024). TERT promoter mutations contribute to adverse clinical outcomes and poor prognosis in radioiodine refractory differentiated thyroid cancer. Sci. Rep..

[30] Hong C.M., Ahn B.C. (2017). Redifferentiation of radioiodine refractory differentiated thyroid cancer for reapplication of I-131 therapy. Front. Endocrinol..

[31] Na H.Y., Yu H.W., Kim W., Moon J.H., Ahn C.H., Choi S.I., Kim Y.K., Choi J.Y., Park S.Y. (2022). Clinicopathological indicators for TERT promoter mutation in papillary thyroid carcinoma. Clin. Endocrinol..

[32] Aashiq M., Silverman D.A., Na’ara S., Takahashi H., Amit M. (2019). Radioiodine-refractory thyroid cancer: Molecular basis of redifferentiation therapies, management, and novel therapies. Cancers.

[33] Yu P., Qu N., Zhu R., Hu J., Han P., Wu J., Tan L., Gan H., He C., Fang C. (2023). TERT accelerates BRAF mutant-induced thyroid cancer dedifferentiation and progression by regulating ribosome biogenesis. Sci. Adv..

